# Supplementary material for: Evaluation of Right Ventricular Function and Myocardial Microstructure in Fetal Hypoplastic Left Heart Syndrome
Source: J Clin Med. 2022 Jul 30;11(15):4456. doi: 10.3390/jcm11154456 (PMC9369849; doi:10.3390/jcm11154456)
Supplement: Supplementary file 1 [file jcm-11-04456-s001.zip › jcm-1762639-supplementary.pdf]

supplemental material

Table S1. Correlation between HA degrees and RVGLS (%) in HLHS fetuses.

Variable	R^2	P value
Basal		
lateral	0.97	0.02
anteroseptal	0.83	0.09
inferoseptal		
Middle		
lateral	0.71	0.16
anteroseptal	0.67	0.39
inferoseptal		
Apical		
lateral	0.12	0.65
anteroseptal		
inferoseptal		

HA, helix angle, 95%CI, 95% confidence interval.

Table S2. Correlation between FA, MD and RVGLS in HLHS fetuses.

Variable	R^2	P value
FA	0.0028	0.95
MD	0.93	0.04

FA, fractional anisotropy, MD, mean diffusivity.

Table S3. Inter-Observer and Intra-Observer Reproducibility for the Parameters of Myocardial deformation.

	ICC (95% CI)	Bias	95% LOA
Inter-observer (n=30)			
RVGLS (%)	0.85(0.81-0.93)	0.26	-3.69-4.52
RVGLVs (cm/s)	0.86(0.83-0.92)	0.09	-0.36-0.40
RVGLSRs(s^{-1})	0.82(0.79-0.88)	-0.12	-0.52-0.36
RVGLDd (mm)	0.88(0.81-0.90)	-0.09	-0.82-0.79
RVGLVd (cm/s)	0.86(0.79-0.92)	0.13	-1.06-1.10
RVGLSRd(s^{-1})	0.84(0.76-0.89)	0.16	-1.23-1.68
Intra-observer (n=30)			
RVGLS (%)	0.90(0.82-0.96)	0.04	-2.99-2.44
RVGLVs (cm/s)	0.85(0.78-0.89)	0.02	-0.31-0.35
RVGLSRs(s^{-1})	0.88(0.80-0.92)	-0.01	-0.39-0.31
RVGLDd (mm)	0.91(0.83-0.96)	-0.02	-0.45-0.43
RVGLVd (cm/s)	0.89(0.81-0.91)	0.10	-1.25-1.45
RVGLSRd(s^{-1})	0.91(0.84-0.95)	0.05	-0.59-0.69

RVGLS, right ventricle global longitudinal strain. RVGLV, right ventricle global longitudinal velocity, RVGLD, right ventricle global longitudinal displacement, RVGLSR, right ventricle global longitudinal strain rate. 95% LOA, 95% limits of agreement.
